# Identification of functional *cis*-acting RNA elements in the hepatitis E virus genome required for viral replication

**DOI:** 10.1371/journal.ppat.1008488

**Published:** 2020-05-20

**Authors:** Xiaohui Ju, Guangtao Xiang, Mingli Gong, Rui Yang, Jierui Qin, Yafei Li, Yuchen Nan, Yonglin Yang, Qiangfeng Cliff Zhang, Qiang Ding

**Affiliations:** 1 Center for Infectious Disease Research, School of Medicine, Tsinghua University, Beijing, China; 2 School of Life Sciences, Tsinghua University, Beijing, China; 3 Department of Preventive Veterinary Medicine, College of Veterinary Medicine, Northwest A&F University, Yangling, Shaanxi, China; 4 Department of General Practice, Nanjing First Hospital, Nanjing Medical University, Nanjing, China; 5 Beijing Advanced Innovation Center for Structural Biology, Tsinghua University, Beijing, China; The Research Institute at Nationwide Children's Hospital, UNITED STATES

## Abstract

There are approximately 20 million events of hepatitis E virus (HEV) infection worldwide annually. The genome of HEV is a single-strand, positive-sense RNA containing 5’ and 3’ untranslated regions and three open reading frames (ORF). HEV genome has 5’ cap and 3’ poly(A) tail to mimic host mRNA to escape the host innate immune surveillance and utilize host translational machineries for viral protein translation. The replication mechanism of HEV is poorly understood, especially how the viral polymerase distinguishes viral RNA from host mRNA to synthesize new viral genomes. We hypothesize that the HEV genome contains *cis*-acting elements that can be recognized by the virally encoded polymerase as “self” for replication. To identify functional *cis*-acting elements systematically across the HEV genome, we utilized an ORF1 transcomplementation system. Ultimately, we found two highly conserved *cis*-acting RNA elements within the ORF1 and ORF2 coding regions that are required for viral genome replication in a diverse panel of HEV genotypes. Synonymous mutations in the *cis*-acting RNA elements, not altering the ORF1 and ORF2 protein sequences, significantly impaired production of infectious viral particles. Mechanistic studies revealed that the *cis*-acting elements form secondary structures needed to interact with the HEV ORF1 protein to promote HEV replication. Thus, these *cis*-acting elements function as a scaffold, providing a specific “signal” that recruits viral and host factors to assemble the viral replication complex. Altogether, this work not only facilitates our understanding of the HEV life cycle and provides novel, RNA-directed targets for potential HEV treatments, but also sheds light on the development of HEV as a therapeutic delivery vector.

## Author summary

Hepatitis E virus (HEV) is an underestimated pathogen, causing approximately 20 million infections worldwide annually and leading to about 60,000 deaths. There are no direct-acting antivirals for treating HEV, and although significant progress has been made to establish robust HEV cell culture models, the life cycle remains poorly characterized. A better understanding of HEV replication could facilitate the development of new drugs targeting this critical process. Our study found that RNA elements in the HEV genome interact with the HEV replicases to promote viral replication, suggesting that these RNA elements function as a scaffold for recruitment and assembly of the viral replication complex. This work furthers our understanding of HEV replication and could inform the generation of RNA-based therapeutics for treating HEV infection.

## Introduction

Hepatitis E virus (HEV) remains a considerable health problem in both developing and developed countries, causing an estimated 60,000 deaths and 3,000 stillbirths a year [1, 2]. In healthy individuals, HEV infections are usually self-limiting. However, HEV can establish chronic infections and induce liver cirrhosis in immunosuppressed patients, such as organ-transplant recipients and individuals infected with human immunodeficiency virus (HIV) [3, 4]. Although the overall fatality rate of HEV infection in the general population is about 1%, in pregnant women, it can be an extremely serious illness with a mortality rate of up to 30% during the third trimester [5, 6]. The mechanisms underlying this severe pathogenesis remain incompletely understood. The HEV genome was first cloned in 1990 [7], and significant progress has been made to establish animal and cell culture models to study the HEV life cycle and pathogenesis [8, 9]. However, many important questions about HEV biology are still not well elucidated.

HEV is a positive-sense, single-stranded RNA virus belonging to the *Hepeviridae* family and the *Orthohepevirus* genus, which is subdivided into four species (A, B, C, and D). The HEV strains infecting humans all belong to the *Orthohepevirus A* species and include genotypes 1, 2, 3, 4, and 7 [1, 10]. The HEV RNA genome is approximately 7.2 kb in length, and its three open reading frames (ORFs) are flanked by a 5’ and a 3’ untranslated region (UTR). ORF1 is a nonstructural polyprotein comprised of a methyltransferase [11, 12], Y domain [13], putative papain-like cysteine protease [14–16], hypervariable region (HVR) [17, 18], polyproline region [19], X domain [20, 21], RNA helicase [22–24], and RNA-dependent RNA polymerase (RdRp) [1, 7, 25]. It remains controversial whether ORF1 functions as a polyprotein with multiple domains or is instead processed by its putative protease domain into individual proteins during the HEV life cycle [26, 27]. Recently, a recombinant HEV harboring epitope tags in the ORF1 protein was generated, and no processed products of ORF1 were observed during HEV replication [28], suggesting that ORF1 can function as a polyprotein to replicate the viral genome. ORF2 encodes the viral capsid and is involved in virion assembly and interaction with the putative host receptor to mediate virion entry [7, 29]. ORF3 is a viroporin that is essential for release of infectious particles from infected cells [30, 31].

After entering hepatocytes, HEV can translate ORF1 directly from its RNA genome [32, 33]. Furthermore, the viral RNA genome is used by ORF1 to synthesize the antigenomic RNA, which functions as the template for generating more of the positive-sense viral RNA genome by ORF1 [34, 35]. Meanwhile, from a promoter in the antigenomic RNA, ORF1 transcribes the subgenomic RNA from which the ORF2 and ORF3 proteins are then translated [35, 36]. The progeny viral RNA genomes are in turn recognized by ORF2 for packaging into viral particles that are subsequently released from the cell [37]. Hence, to fulfill these multiple functions, the HEV RNA genome must form secondary or higher-order structures as specific signals (*cis*-acting RNA elements) for this complex process to be successfully completed. These RNA structures can be involved in interactions with viral and cellular proteins during viral genome translation [38], replication and encapsidation [39]. It has been found that the highly structured 5′- and 3′ UTRs of the HEV genome contains essential *cis*-acting RNA elements that can also extend into adjacent coding sequences [40, 41].

RNA structures or *cis*-acting RNA elements are critical for the life cycles of multiple RNA viruses [42, 43] and have been identified by using RNA folding prediction programs and classical comparative phylogenetic analyses of viral genome sequences [44–47]. The functional significance of the resultant secondary structure models can then be validated by reverse genetics approaches such as site-directed mutagenesis. Recently, substantial effort has been made to develop high-throughput approaches for analyzing RNA secondary structure. Selective 2′-Hydroxyl Acylation analyzed by Primer Extension (SHAPE) is one such method that discovered a high-resolution model for an HIV-1 RNA genome [48, 49].

An HEV ORF1 transcomplementation cell culture system, where HepG2C3A hepatoma cells are lentivirally transduced with HEV ORF1, was recently established as an efficient tool for understanding HEV replication and transcription [36]. In this system, the transduced ORF1 can function *in trans*, providing the polymerase activity needed to successfully replicate transfected HEV RNA. This ORF1 transcomplementation system thus creates a unique opportunity to uncouple viral RNA function from protein translation. Using this tool, the promoter regulating the HEV subgenomic RNA, from which OR2 and ORF3 are translated, was identified [36].

In this study, we performed an unbiased screen across the entire HEV genome using this ORF1 transcomplementation system and identified two *cis*-acting RNA elements in the ORF1 and ORF2 coding region of the KernowC1/p6 strain (genotype 3; nucleotides 102–131 and 7311–7340, respectively). These two *cis*-acting elements are highly conserved across HEV genotypes and required for genome replication of multiple HEV genotypes. Furthermore, mechanistic studies found that these *cis*-acting elements form secondary RNA structures that recruit ORF1 and subsequent assembly of the viral replication complex.

Collectively, we describe two novel, essential *cis*-acting elements in the HEV genome and highlight the interplay of RNA structure with viral replicases to dictate viral genome replication. These findings shed more light on our understanding of viral replication and could inform the generation of RNA-based therapeutics for treating HEV infection.

## Results

### Using an ORF1 transcomplementation system to identify functional *cis*-acting RNA elements in the HEV genome required for viral replication

**Fig 1:**
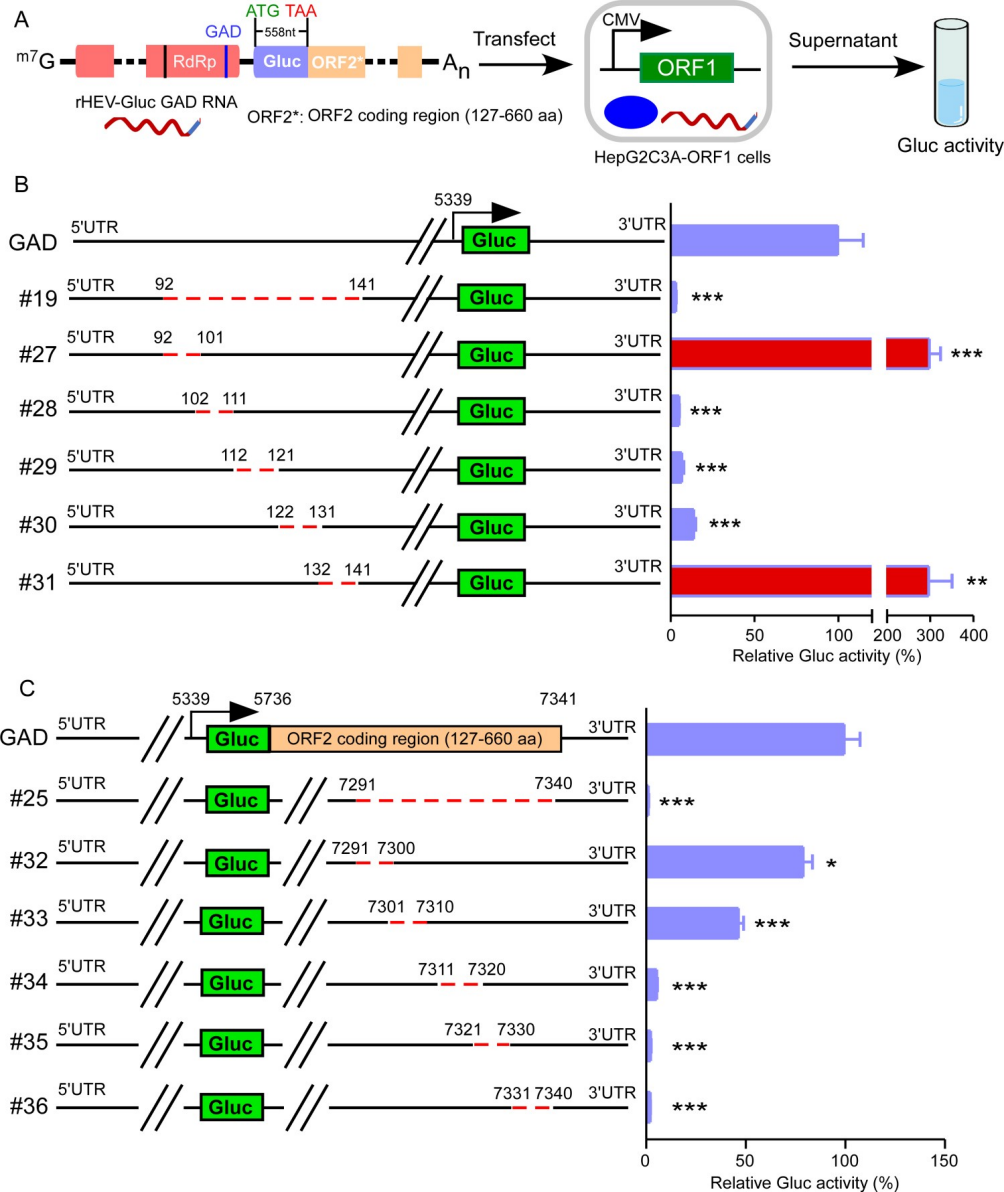
Identification of functional *cis*-acting RNA elements by ORF1 transcomplementation system. (A) Schematic representation of how *cis*-acting RNA elements were identified by utilizing an HEV replicon and ORF1 transcomplementation system. (B) and (C) Replication of HEV RNA mutants in ORF1-transcomplemented HepG2C3A cells. A series of truncated HEV RNA replicons harboring a secretory Gaussia luciferase (Gluc) reporter were transfected into HepG2C3A-ORF1 cells. Cell culture supernatants from each group were collected and Gluc activity measured two days post-transfection. The data are presented as the percentage of Gluc activity relative to that of the full-length rHEV-Gluc GAD. #27 and #31 mutants’ replication were enhanced, as indicated by increased Gluc activities (red). Values are means plus standard deviations (SD) (error bars) (n = 4). *, P < 0.05; **, P < 0.01; ***, P < 0.001. Significance assessed by one-way ANOVA.

HEV replication involves complex interactions between the viral genome and viral replicase as well as host factors [50]. To gain more insights into the HEV replication mechanism, we sought to identify *cis*-acting RNA elements in the viral genome required for HEV replication. We previously established an HEV ORF1-based transcomplementation cell culture model and demonstrated that ORF1 could function *in trans* to replicate the viral genome [36]. Using this system with an HEV replicon encoding a secretory Gaussia luciferase (Gluc) reporter [41], we uncouple the HEV RNA from ORF1 protein coding function. This allowed us to perform systematically an unbiased screen for functional *cis*-acting RNA elements required for HEV replication across the whole HEV genome, excluding the 5’ and 3’UTRs (Fig 1A).

First, we generated a series of truncated Kernow C1/p6-Gluc GAD (rHEV-Gluc GAD) replicon mutants harboring several hundred nucleotide (nt) deletions between positions 27nt to 4766nt and positions 5741nt to 7340nt (S1 Fig). These mutant RNAs were *in vitro* transcribed and subsequently transfected into lentiviral transduced HepG2C3A cells stably expressing Kernow C1/p6 ORF1 (HepG2C3A-ORF1) [36], and the Gluc activity of the supernatant was monitored 2 days post-transfection. Although most deletions did not affect Gluc activity, some deletions had a slight or moderate effect (~10%-50% reduction) on Gluc activity compared to the full-length rHEV-Gluc GAD (S1 Fig, #3, #6, #12 and #13). These results suggested that the deletions we generated did not cause the general alteration of HEV genome structure to disrupt HEV replication. Notably, deleting 27nt to 241nt or 7141nt to 7340nt (S1 Fig, #1 or #16) reduced Gluc activity to a level similar to that of the junction region depleted (ΔJR) [41, 51] mutant. These observations suggested that functional *cis*-acting RNA elements required for HEV replication were likely contained in these regions.

To accurately localize the relevant sequences, we created additional mutant rHEV-Gluc GAD replicon genomes lacking 50-nt segments between positions 27nt to 241nt or 7141nt to 7340nt (S2 Fig). As before, these replicon genomes were transfected into HepG2C3A-ORF1 cells, and the Gluc activity of the supernatants assessed. Only the deletion of 92nt-141nt or 7291nt-7340nt phenotypically copied the larger deletion of 27nt-241nt or 7141nt-7340nt, respectively (S2 Fig, #1 *vs* #19; #16 *vs* #25). To more accurately pinpoint the functional *cis*-acting RNA elements, we generated series of 10nt deletions between 92nt-141nt or 7291nt-7340nt. Deletion of 102nt-111nt, 112nt-121nt or 122nt-131nt dramatically impaired HEV replication to a degree comparable with the deletion of 92nt-141nt (Fig 1B, #19 *vs* #28, #29 or #30). Deletion of 7311nt-7320nt, 7321nt-7330nt or 7331nt-7340nt significantly decreased HEV replication more than 90%, comparable to deletion of 7291nt-7340nt (Fig 1C, #25 *vs* #34, #35 or #36). Intriguingly, we also noticed that some deletions, 92nt-101nt or 132nt-141nt, enhanced HEV replication by 3-fold (Fig 1B, GAD *vs* #27 or #31), suggesting the presence of RNA elements that negatively regulate virus replication. Collectively, these data suggest that viral *cis*-acting RNA elements can function as positive or negative regulators of viral genome replication. Of note, nucleotides 102–131 (within the methyltransferase domain of ORF1) and 7311–7340 (ORF2 coding region) constitute *cis*-acting RNA elements required for HEV replication.

### Validating the requirement of *cis*-acting elements for HEV replication in an HEV replicon model

**Fig 2:**
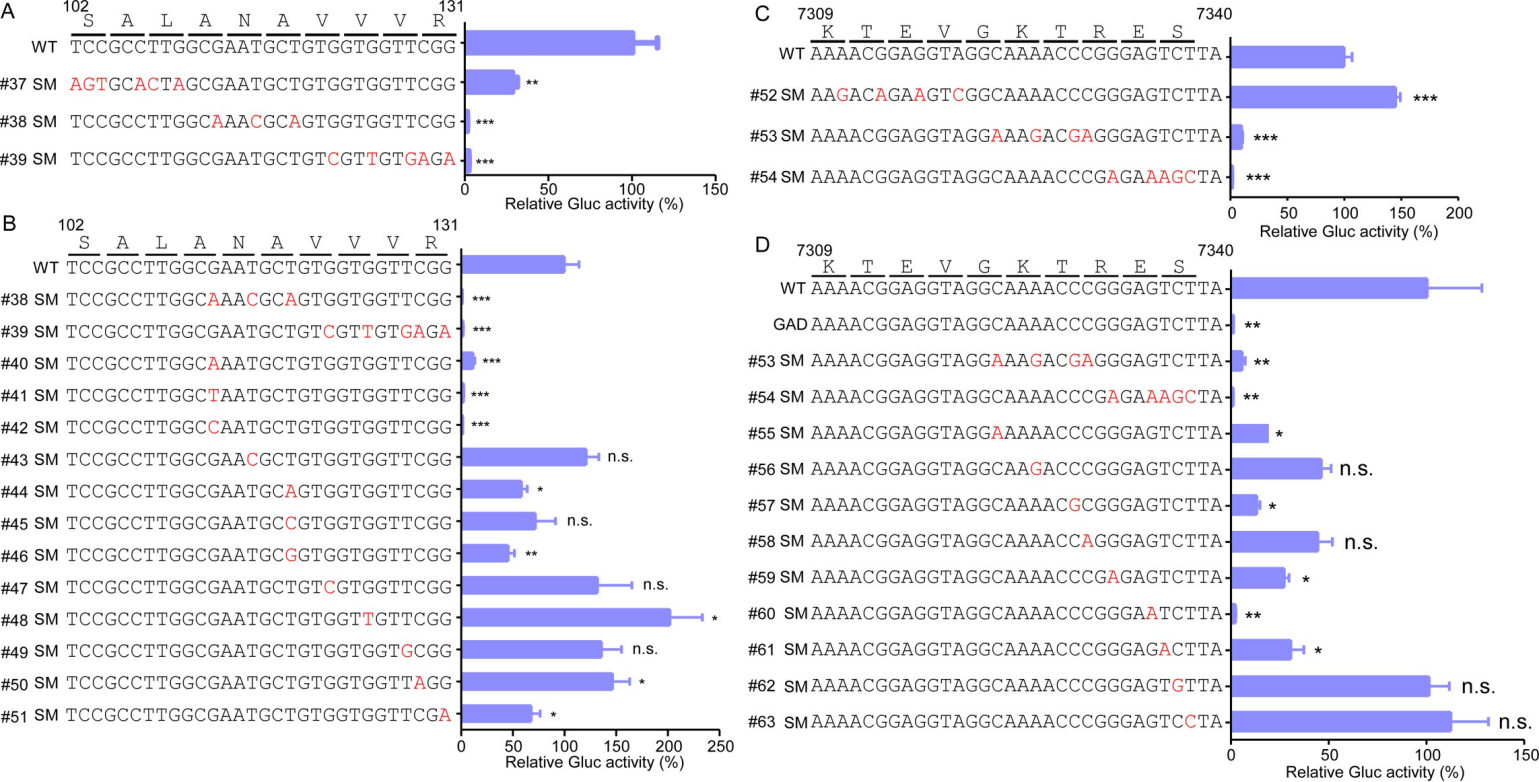
The synonymous mutations in the *cis*-acting RNA elements severely impair HEV replication. Synonymous mutations were introduced in the (A-B) ORF1 (102-131nt) or (C-D) ORF2 (7309-7340nt) coding regions. rHEV-Gluc WT, synonymous mutants, or GAD mutant replicon RNA were transfected into HepG2C3A cells. Cell culture medium was collected two days after transfection and Gaussia luciferase activity quantified. The data are presented as the percentage of Gaussia luciferase activity relative to that of the WT rHEV-Gluc replicon. The numbering denotes the positions of the Kernow C1/p6 viral genome. Values are means plus SD (n = 4). *, P < 0.05; **, P < 0.01; ***, P < 0.001; n.s., not significantly different by one-way ANOVA.

**Fig 3:**
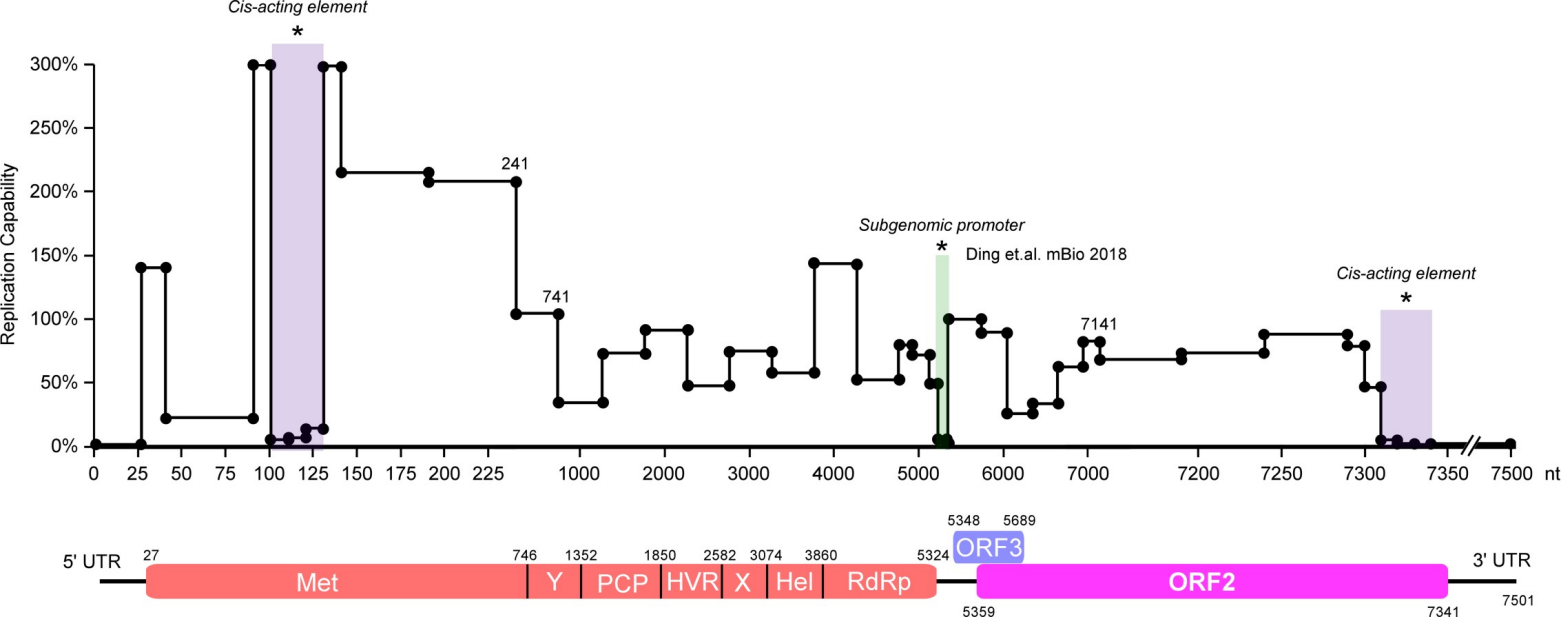
Localization of functional *cis*-acting elements in the HEV genome. The effect of sequences in the HEV genome on HEV replication capability is summarized based on the data in Figs 1 and 2, S1 and S2 Figs in this study (highlighted as purple) and published results (green) [35]. The Y axis shows the relative level of viral replication as determined in the viral replicon system when the corresponding region (shown on the X axis) in the HEV genome was deleted.

Next, we aimed to validate the importance of these novel *cis*-acting RNA elements during HEV replication with an HEV subgenomic replicon. We introduced synonymous mutations every 3 or 4 amino acids in the 102nt-131nt region of the ORF1 sequence of Kernow C1/p6-Gluc (rHEV-Gluc) (Fig 2A). The *in vitro*-transcribed WT or synonymous mutant (SMs) viral replicon RNA were transfected into HepG2C3A cells, and Gluc activity was monitored to measure as a proxy for replication. Gluc activity was dramatically reduced in the supernatants of cells transfected with the rHEV-Gluc SMs compared to the parental sequence. Notably, #38 and #39 SM mutants harboring 3 and 5 mutations, respectively, were severely impaired, with a 95% reduction in Gluc activity compared to the WT replicon (Fig 2A). To determine which of the nucleotides within this region are particularly important, we created additional mutant replicons, each containing a single nucleotide mutation (Fig 2B, #40-#51). Gluc activity was reduced to levels comparable to that of the #38 SM mutant in only single SM mutants at residue 113 (G113A, G113T or G113C; Fig 2B, #38 *vs*.*#*40-#42), suggesting that G113 is critical for HEV replication. Similarly, we took the same approach in the region 7309–7340 nt of ORF2 and identified G7335 as the particularly important nucleotide for HEV replication (Fig 2C and 2D, #60). Collectively, our analysis identified two novel *cis*-acting RNA elements (102nt-131nt and 7311nt-7340nt) in the HEV genome (Fig 3), with nucleotides G113 and G7335 particularly important as demonstrated by the drastic impairment in replication upon changing these single nucleotides.

### The *cis*-acting RNA elements are highly conserved and required for pan-genotype HEV replication

**Fig 4:**
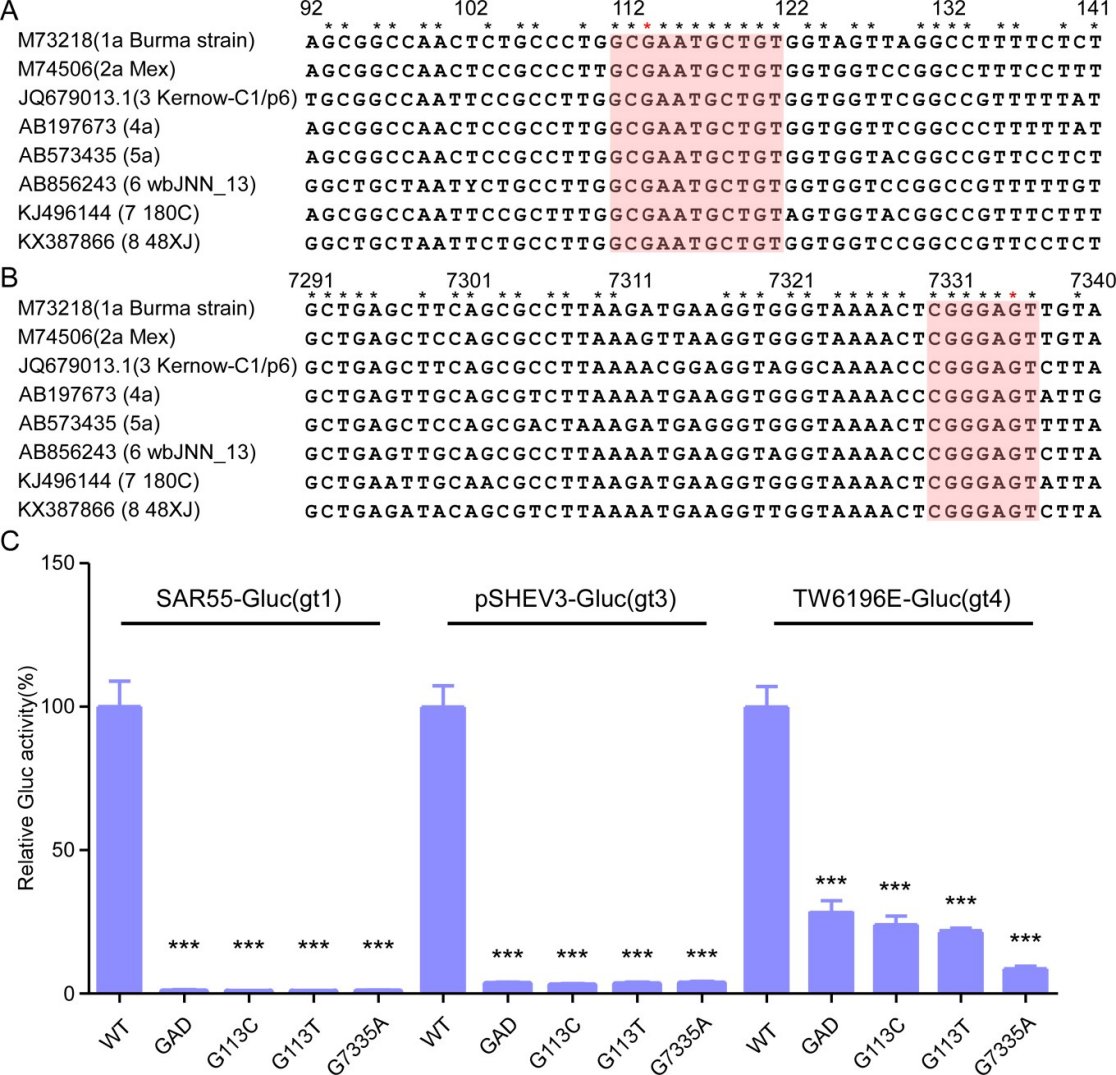
The *cis*-acting RNA elements are conserved and functional in multiple HEV genotypes. (A-B) Consensus sequences of the *cis*-acting RNA elements in ORF1 (A) and ORF2 (B) across genotypes 1 to 8 were aligned by MEGA6 software. The consensus sequences are shaded. G113 and G7335 are highlighted with a red star. (C) WT, synonymous mutant (G113C, G113T or G7335A) or GAD mutant RNA of SAR55 (gt1), pSHEV3 (gt3) or TW6196E (gt4) were transfected into HepG2C3A cells. Cell culture medium was collected two days after transfection, and Gaussia luciferase activity quantified. The data are presented as the percentage of Gaussia luciferase activity relative to that of the WT rHEV-Gluc. The numbering denotes the positions of the Kernow C1/p6 viral genome. Values are means plus SD (n = 4). ***, P < 0.001 by one-way ANOVA.

We then aimed to validate the importance of these *cis*-acting RNA elements in other genetically diverse HEV genotypes. First, we assessed the conservation of these regions in the other genotypes of the *Orthohepevirus A* species. Sequence alignments of the *cis*-acting elements and their proximal regions (with the Kernow C1/p6 viral genome as our reference) showed that, compared to the proximal regions, the 111nt-121nt and 7330nt-7336nt are highly conserved (100%) across genotypes 1 to 8 (Fig 4A and 4B), which is consistent with the idea that these segments are critical for an essential step in HEV replication. We extended our analysis to other *Hepeviridae* species: *Orthohepevirus B*, *C*, and *D* of the *Orthohepevirus* genus and *Piscihepevirus A* of the *Piscihepevirus* genus (S3 Fig). The *cis*-acting elements were exclusively conserved in *Orthohepevirus A* species, suggesting that other *Orthohepevirus* species and members of the *Piscihepevirus* genus evolved different mechanisms of viral genome replication.

To determine if the conserved regions were also critical to viral replication of other *Orthohepevirus A* genotypes (GTs), we introduced synonymous mutations in the ORF1 (G113C or G113T) or ORF2 (G7335A) coding sequences of SAR55-Gluc (GT1) [52], pSHEV3-Gluc (GT3) [53], and TW6196E-Gluc (GT4) [54] replicons (Fig 4C). The *in vitro* transcribed WT, GAD or SM replicon RNA for each of these replicons was transfected into HepG2C3A cells, and Gluc activity was measured. Consistent with previous data, Gluc activity was reduced in the supernatants of cells transfected with the SMs G113C, G113T and G7335A) at levels comparable to those of the GAD mutant (Fig 4C and S4 Fig). These results suggest that the novel *cis*-acting RNA elements we identified here are highly conserved across HEV genotypes and are critical for pan-genotype HEV replication.

### The *cis*-acting RNA elements are required for HEV virus infectivity

**Fig 5:**
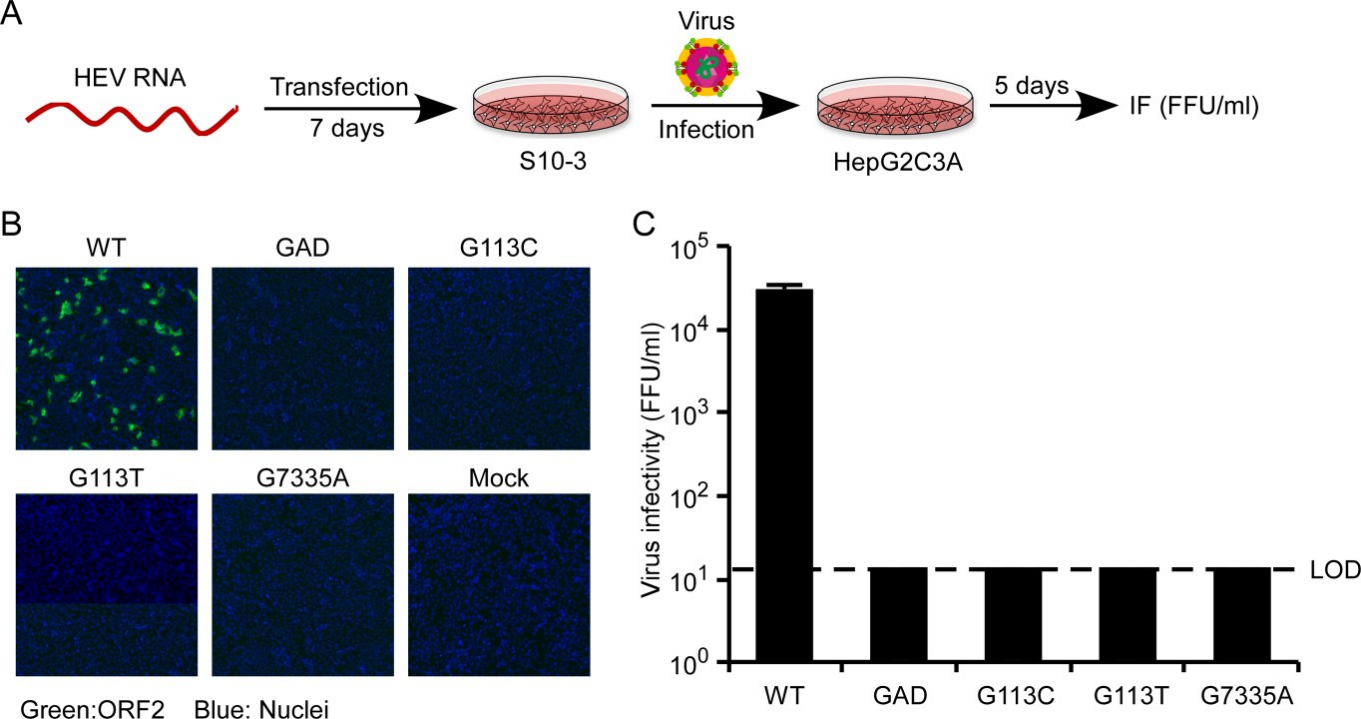
Full-length HEV genome with synonymous mutations in the *cis*-acting RNA elements is significantly impaired in its ability to produce infectious virus. (A) Schematic diagrams of the protocol to determine the production of infectious virus. For the *cis*-acting element mutants, synonymous mutations were introduced in the ORF1 coding region (G113C or G113T) or ORF2 coding region (G7335A). (B-C) Transfection of *in vitro*-transcribed WT, synonymous mutants or GAD (Pol-) RNA of full-length Kernow C1/p6 (GT 3) into S10-3 cells. Cell lysate supernatant was collected 7 days after transfection, and virus was titrated by infecting HepG2C3A cells. Cells were stained with anti-HEV ORF2 mAbs at 5 days post of infection. (B) to quantify infectious viral particles by foci-forming assay (C). Values are means plus SD (n = 3). IF, immunofluorescence; FFU, foci-forming units; LOD, limit of detection.

To validate the importance of *cis*-acting elements in the context of a full-length infectious viral genome, we introduced the same SMs in the Kernow C1/p6 genome while retaining the ORF1 and ORF2 protein sequence (Fig 5A). The *in vitro* transcribed RNAs of the parental Kernow C1/p6 and SM genomes were transfected into the human hepatoma cell line S10-3 [55]. At day 7 post-transfection, the cells were lysed and the virus in lysate supernatant was titrated on naïve HepG2C3A cells using an HEV ORF2 antibody (2G8). About 3×10^4^ FFU/ml of HEV was detected in the lysate supernatant collected from S10-3 cells transfected with the parental genome whereas no infectious particles were detected for the SM mutants or GAD genome (Fig 5B and 5C). These data further affirm that positions 102–131 nt and 7311–7340 nt of the HEV genome harbor a region critical for viral genomic RNA synthesis and ultimately production of infectious virions.

### RNA structure in the HEV genome interacts with the ORF1 protein to facilitate viral replication

Synonymous mutations in genes do not alter the encoded protein sequences, but codon usage biases can affect gene expression and have been observed in eukaryotic and prokaryotic genomes [56–58]. To exclude the possibility that the decreased HEV replication of the full-length SM genomes was a consequence of inefficient ORF1 expression caused by the SMs, we transduced the WT and SM ORF1 cDNAs under the control of a CMV promoter into HepG2C3A cells and assessed ORF1 protein levels by western blot. WT and SM ORF1 protein levels were comparable (S5A Fig), indicating that the synonymous mutations do not affect ORF1 expression. In addition, we detected only unprocessed ORF1 protein (~190 kD), which suggests that the ORF1 was not processed in our experimental system. This result was consistent with a recent finding using a recombinant hemagglutinin (HA) epitope-tagged HEV replicon system [28].

**Fig 6:**
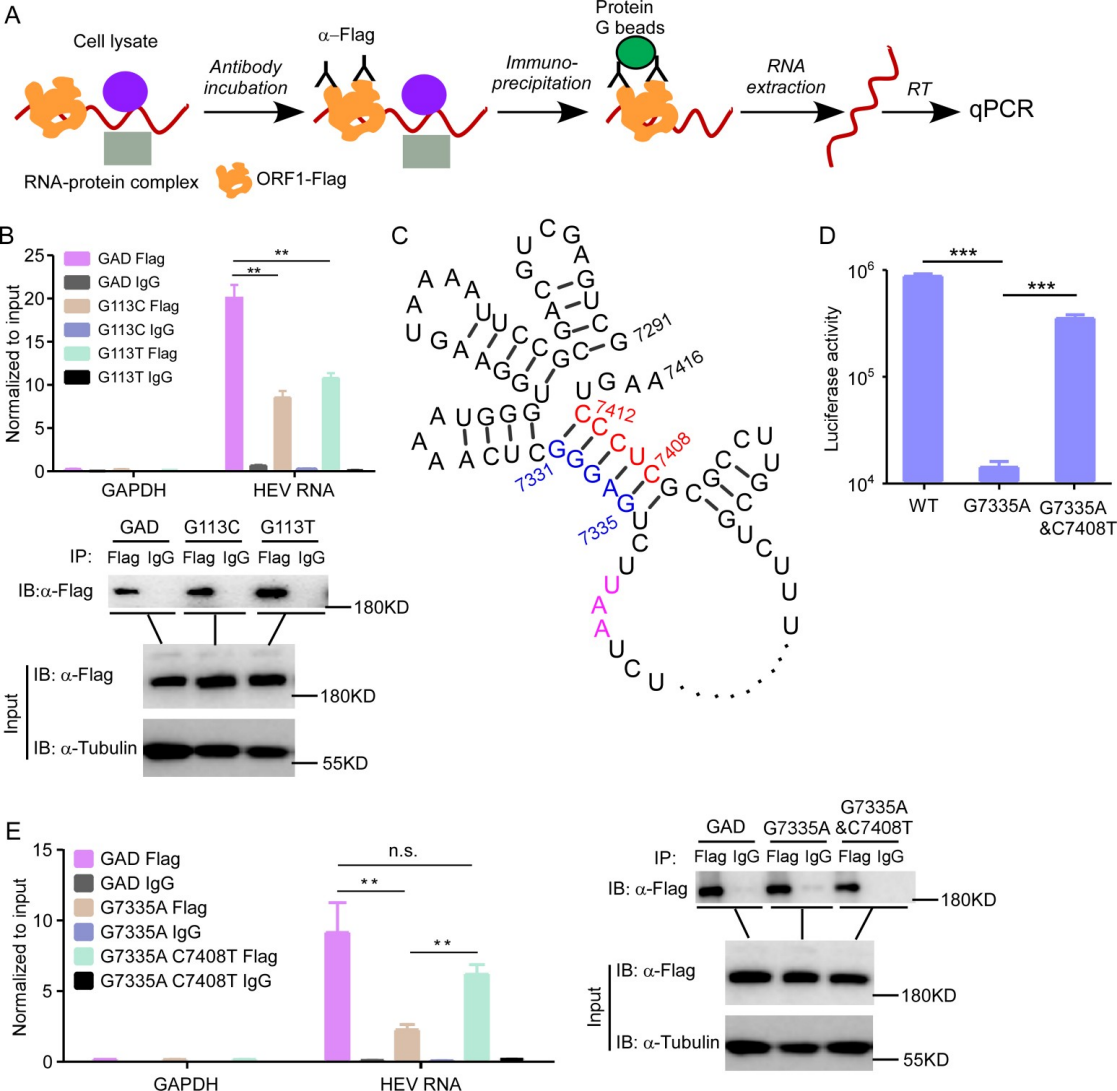
The *cis*-acting RNA elements interact with HEV ORF1. (A) A schematic representation of the RNA immunoprecipitation (RIP) assay used to examine the interaction of the HEV RNA genome with the ORF1 protein. (B) The 293T cells overexpressing ORF1 (GAD)-Flag were transfected with GAD or synonymous mutants of the viral genome. The cells were lysed and then incubated with Flag antibody to perform RIP with lgG as the negative control. The immunoprecipitated complex was subjected to RNA purification, and the ORF1-associated RNA were detected by RT-qPCR analysis. Enrichment of RNA binding to ORF1 is shown as fold normalized to input. Immunoblotting analysis was performed to confirm the efficacy of ORF1 (GAD)-Flag immunoprecipitation. GAPDH, glyceraldehyde-3-phosphate dehydrogenase. (C) RNA structure prediction of the secondary structures of *cis*-acting RNA elements. RNAalifold [45] (http://rna.tbi.univie.ac.at/cgi-bin/RNAWebSuite/RNAalifold.cgi) was utilized to predict the secondary structures, suggesting that the G7335 would match C7408 to form the stem structure. (D) C7408T mutation restored the replication of the G7335A mutant. To verify the role of the stem structure in HEV replication based on this analysis, C7408T would restore the stem structure impaired in the G7335T mutant. Therefore, the C7408T mutation was introduced into G7335T mutant and the viral replicon RNA harboring the two mutations was transfected into HepG2C3A cells. The Gluc activity was measured as described previously. (E) RIP assay to analyze the association of G7335A mutant or G7335A/C7408T double mutant with HEV ORF1. This assay was performed as described in (B). Values are means plus SD (n = 3). **, P < 0.01; ***, P < 0.001; n.s., not significantly different by Student’s t test or one-way ANOVA.

We hypothesized that the *cis*-acting RNA elements form secondary structures that interact with HEV ORF1 and are thus essential for virus replication. To determine the HEV RNA interaction with HEV ORF1, we conducted an RNA immunoprecipitation (RIP) assay [59, 60]. We transfected Kernow C1/p6 HEV GAD or SM mutants (G113C or G113T) positive-strand viral RNA genome into 293T cells expressing HEV ORF1 (GAD)-Flag. Cells were lysed and incubated with Flag antibody to capture the viral replication complex. These immunoprecipitated lysates were washed and then subjected to RNA extraction. The purified RNA was analyzed by RT-qPCR assay to determine HEV RNA abundance (Fig 6A). ORF1 did specifically interact with HEV RNA but not host GAPDH mRNA. Compared to the GAD mutant, the G113C or G113T mutants had reduced ORF1 binding (approximately 50%-60%) (Fig 6B), which is consistent with our functional experiments that showed these mutations significantly impaired HEV replication. Additionally, an RNA pulldown confirmed that the G113C and Δ92–141 mutants were impaired in binding ORF1 with approximately 50%-60% reduction (S5B and S5C Fig). It is conceivable that other *cis*-acting elements, such as 5’UTR, 3’UTR, subgenomic RNA promoter, etc. also contribute to the binding with ORF1 in the G113C or G113T mutant genome, but are not sufficient for viral replication. Notably, G113C or G113T mutations in the negative-strand HEV RNA genome had little effect on ORF1 binding compared with GAD (S6A Fig). These data suggest that the G113C or G113T mutations significantly decrease the interaction of ORF1 with specifically the positive-strand viral RNA, thus severely impairing the synthesis of negative-strand viral RNA (S6B and S6C Fig).

We speculated that the *cis*-acting elements formed RNA structures required for interaction with ORF1. However, we could not validate functional structures in the 92-141nt regions (S7A–S7D Fig). Of note, a highly conserved RNA structure was predicted between the C-terminal of ORF2 and the 3’UTR among 8 HEV genotypes. The 7331-7336nts in ORF2 were predicted to base pair with 7407-7412nts within the 3’UTR to form a stem, and a G7335A mutation could potentially disrupt this stem structure and thus impair HEV replication (Fig 6C). To examine the potential role of this stem structure, we engineered a C7408T complementary mutation which would theoretically restore the secondary structure of our G7335A mutant. The complementary mutation partially rescued replication (Fig 6D), demonstrating that the stem structure is required for HEV replication. Consistently, the RIP experiment suggested that the G7335A mutation severely disrupts the interaction of viral RNA with ORF1 but could be significantly rescued by the complementary mutation C7408T (Fig 6E). Collectively, these results demonstrate that the novel *cis*-acting RNA elements we identified are important and specific signals for recruiting ORF1 for viral genome replication. Mutations that disrupt these elements could dramatically impair virus replication.

## Discussion

Previous studies showed that the 5’UTR and 3’UTR contain *cis*-acting elements required for HEV genome replication [40, 41]. In addition, a single synonymous mutation in ORF2 (7106nt of the SAR55 strain) was found to alter HEV virulence *in vivo* [52]. Another study identified two stem-loop RNA structures in the central region of ORF2 that are required for viral replication by analyzing the conservation of the synonymous sites in HEV sequence alignments [61]. However, comprehensive, function-based identification of *cis*-acting RNA elements in the HEV genome are necessary to further understand the mechanism governing viral replication. In this study, we performed an unbiased approach to screen for *cis*-acting RNA elements in the ORF regions and identified two novel *cis*-acting elements in the ORF1 and ORF2 coding region that are required for HEV replication. We used the Kernow C1/p6 (genotype 3) replicon, which harbors the S17 insertion in the ORF1 [62, 63], as the reference for our screen. Intriguingly, we did not observe a dramatic decrease in HEV replication after removal of the S17 insertion (S1A Fig, #6), indicating that the enhancement of HEV replication by this insertion is due to alteration of the ORF1 protein rather than the RNA structure. The previous reported *cis*-acting elements in ORF2 [61] had a moderate ability to impair HEV replication (S1B Fig, #13), and we only chose to focus on candidates that affected HEV replication by more than 90%. The results of our screen demonstrated that most of the regions in the HEV genome are not essential for HEV replication, suggesting that the HEV genome is flexible and tolerant of manipulation, which could be utilized in developing vectors for therapeutic purposes.

These *cis*-acting elements we identified are highly conserved across all eight HEV genotypes and were required for replication of genetically diverse HEV genomes (GT1, 3 and 4). To ascertain that these sequence elements are functionally relevant for virus infectivity, we mutated these sequences in the context of full-length HEV genomes. Indeed, mutations within the *cis*-acting elements abrogated the production of infectious HEV virions. Since the mutations we introduced were synonymous, the reductions in viral replication can be directly attributed to the disruption of *cis*-acting element, not to any changes in ORF1 expression as demonstrated by western blot. Future structural studies will be crucial to determine how these synonymous mutations affect RNA structure, how ORF1 protein gains specificity for target sequences and a detailed picture of the entire HEV genome’s architecture and secondary structure.

Virus replication is achieved by the viral replication complex, which involves viral RNA/RNA interactions, RNA and viral factor interactions, as well as interaction with host factors [64]. The *cis*-acting RNA elements are specific sequences the viral replicase and related host factors can recognize to initiate the replication complex [65, 66]. Therefore, although viral RNA is only a small fraction of the total RNA in a cell, the viral RNA has a significant advantage to ensure that viral RNA, instead of host RNA, was replicated. However, *cis*-acting RNA elements in the HEV genome are difficult to study since the RNA also serves as the template for translation of gene products, including those required for virus replication. As a result, we believed that our ORF1 based transcomplementation cell culture system was especially well-suited for overcoming this problem. This system was previously utilized to identify the intragenomic promoter directing HEV subgenomic RNA synthesis [36], and we used it here to successfully identify the *cis*-acting elements across the entire viral genome required for HEV replication. Our studies provide an experimental framework to identify the functional *cis*-acting RNA elements regulating viral replication more comprehensively and in greater detail than previously possible. Additionally, ORF1 has also been reported as the genetic determinant of HEV host tropism [67], and ORF1 is the core component of HEV replication complex. It is conceivable that the ORF1 transcomplementation system could be utilized as a tool to further understand HEV host tropism and genetically dissect the HEV replication complex.

To dissect the function of a given viral gene, it is common to introduce mutation(s) into the gene in the infectious clone [68]. However, modifying a coding region could also potentially disrupt the function *cis*-acting elements that reside within the region. Thus, a reliable genomic structural model is necessary to better interpret data and avoid erroneous conclusions. In addition to using reverse genetics to understand viral biology, we can also use these approach to manipulate viral genomes to serve as delivery vectors for foreign genes [68]. However, challenges to this application exist, such as low yields of the recombinant viruses and thus low efficiency of protein production and higher costs. Based on our study, manipulating the viral genome by inserting foreign genes or replacing viral sequences could impair the functional RNA structure and result in the downstream consequences of poor yields. In such cases, it is conceivable that identifying the functional *cis*-acting RNA elements could better inform the design of more efficient viral vectors.

In summary, we identified novel functional *cis*-acting elements in the HEV RNA genome that recruit viral factors to assemble the replication complex for viral genome replication and thus represent a novel RNA-directed drug target. Meanwhile, these *cis*-acting elements serve as the specific signals by which the viral replication complex distinguishes the small fraction of viral RNA from the host RNA to ensure the high-efficiency of replication. Our study not only sheds more light on the mechanisms regulating HEV replication and identifies novel RNA-directed drug targets to combat HEV infection but is also insightful for further development of HEV as a therapeutic delivery vector.

## Materials and methods

### Cell cultures

HEK293T cells (American Tissue Culture Collection, ATCC, Manassas, VA, CRL-3216), HepG2C3A cells (ATCC, CRL-10741) and S10-3 cells were maintained in Dulbecco’s modified Eagle medium (DMEM) (Gibco, NY, USA) supplemented with 10% (vol/vol) fetal bovine serum (FBS), and 50 IU/ml penicillin/streptomycin in a humidified 5% (vol/vol) CO_2_ incubator at 37°C.

### Plasmid construction

The pKernow-C1 p6/Gluc was used as the template to generate the truncation or synonymous mutants for the mapping the functional *cis*-acting RNA elements by ClonExpress MultiS One Step Cloning Kit (C113, Vazyme).

### Lentivirus production

Vesicular stomatitis virus G protein (VSV-G) pseudotyped lentiviruses were produced by transient cotransfection of the third-generation packaging plasmids pMD2G (catalog no. 12259; Addgene) and psPAX2 (catalog number 12260; Addgene) and the transfer vector with VigoFect DNA transfection reagent (Vigorous) into HEK293T cells. The medium was changed 12 h post transfection. Supernatants were collected at 36, 60 and 84 h after transfection, pooled, passed through a 0.45-μm filter, aliquoted, and frozen at -80°C.

### *In vitro* transcription assay and viral RNA transfection

HEV Kernow-C1/p6, HEV Kernow-C1/p6-Gluc, and the truncated mutant plasmids were linearized by MluI. pSAR55-Gluc was linearized by BglII, pGEM-9Zf-pSHEV3-Gluc was linearized by XbaI, and pGEM-7Zf(-)-TW6196E and pGEM-7Zf(-)-TW6196E/Gluc were linearized by SpeI. Viral RNAs were transcribed *in vitro* from linearized plasmid using HiScribe T7 antireverse cap analog (ARCA) mRNA kit (New England Biolabs, Ipswich, MA) according to the manufacturer’s instructions. Viral RNA was transfected into HepG2C3A cells or S10-3 cells using TransIT-mRNA transfection reagent (Mirus Bio LLC, Madison, WI) according to the instructions.

### Gaussia luciferase assays

Gaussia luciferase activity was measured using Renilla Luciferase Assay System (Promega, E2820). Specifically, 10 μl cell culture medium was added per well of a 96-well polystyrene microplate (Corning, NY, USA), followed by the addition of Renilla luciferase assay substrate and the detection of luminescence was performed using Micro plate spectrophotometer (EnVision).

### HEV production and infection

S10-3 cells were seeded into 6-well plate one day before transfection at 50% confluence. Cells are transfected with Kernow C1/p6 WT or mutant RNAs and incubated at 34.5°C for 7 days. For virus collection, cells were trypsinized and pelleted in a 1.5-ml tube by centrifugation, liquid was removed. Cells were lysed by adding 0.6 ml sterilized H_2_O and put on ice for 30 min, further lysed by freeze-thawing 3 times. Cellular debris was removed by centrifugation at 13,000r/m for 15 min, and the supernatant was added with 1/10 volume of 10×phosphate-buffered saline (PBS). For virus infection, HepG2C3A cells were seeded into 24-well plate one day before infection at 30% confluence. Cells were infected with virus in 4% PEG8000 culture medium. The virus mixture was removed one day later, and cell culture medium containing 2% DMSO was added, followed by incubation at 34.5°C for 5 days.

### Ethics statement, mice immunization, and cell fusion

The 6-week old *Balb/C* mice were obtained from Dashuo Biotech (Chengdu, Sichuan, China), and the animal protocol was reviewed and approved by the Animal Welfare Committee of Northwest A&F University. All mice were monitored at a daily basis for any clinical sign. All effort was made to minimize the suffering of mice, and mice were euthanized if a humane endpoint was reached according our protocol. Mice were immunized with 100 μg of recombinant HEV-ORF2 179 protein or MBP-fused HEV-ORF1-X-domain (Sar55 strain) with an equal volume of adjuvant for a total of 3 times with an interval of two weeks. The Freund’s Complete Adjuvant (Sigma-Aldrich) was used for the primary immunization, while Freund’s Incomplete Adjuvant (Sigma-Aldrich) was used for the rest. Mice serum was collected on a weekly basis to monitor the serum antibody level by enzyme-linked immunoprecipitation assay (ELISA). Serum collected before immunization was included as the negative control. After the final immunization, 100 μg of recombinant protein without adjuvant was used to boost the mice 4 days before the spleen collection. The mice with the highest antibody titers were used for cell fusion. Cell fusion of spleen cells with S/p20 cell was conducted as previous described [69]. The selected positive clones of hybridoma were subjected to subcloning via limited dilution. The ELISA positive hybridomas was further screening by using Immunofluorescence microscope or western blot with S10-3 cell transfected HEV-RNA(Sar55). The HEV-179 monoclonal antibody (2G8) and monoclonal antibody-anti-ORF1-X were finally selected for further application.

### Immunofluorescence

Cells in plate were fixed with 4% paraformaldehyde at RT for 20 min. The cells were permeabilized in 0.5% triton X-100 for 20 minutes and blocked in 1% BSA for 60 minutes at RT. Cells were stained by ORF2 antibody (2G8) in 1% BSA and incubated at RT for 1 hour, then cells are washed with PBS three times. Goat anti-mouse IgG (H+L) secondary antibody Alexa Fluor 488 conjugate (1:1000 Invitrogen Cat# A11029) diluted in 1% BSA is used to stain cells at RT for 1 hour, then cells are washed with PBS three times. Nucleus is stained by DAPI for 5 minutes at RT, then cells are washed with PBS three times. The stained cells are observed under microscope.

### Western blotting

Sodium dodecyl sulfate-polyacrylamide gel electrophoresis (SDS-PAGE) immunoblotting was performed as follows: After trypsinization and cell pelleting at 2,000 × g for 10 min, whole-cell lysates were harvested in RIPA lysis buffer (50 mM Tris-HCl [pH 8.0], 150mM NaCl, 1% NP-40, 0.5% sodium deoxycholate, and 0.1% SDS) supplemented with protease inhibitor cocktail (Sigma). Lysates were electrophoresed in 12% polyacrylamide gels and transferred onto nitrocellulose membrane. The blots were blocked at room temperature for 0.5 h using 5% nonfat milk in 1× phosphate-buffered saline (PBS) containing 0.1% (v/v) Tween 20. The blots were exposed to primary antibodies anti-ORF1, β-Tubulin (CW0098, CWBIO), Flag (F7425, Sigma) in 5% nonfat milk in 1×PBS containing 0.1% Tween 20 for 2 h. The blots were then washed in 1×PBS containing 0.1% Tween 20. After 1h exposure to HRP-conjugated secondary antibodies and subsequent washes were performed as described for the primary antibodies. Membranes were visualized using the Luminescent image analyzer (GE).

### RNA Immunoprecipitation (RIP) assay

About 1×10^7^ 293T cells were transfected with 10 μg pLVX-ORF1(GAD)-Flag-zsGreen plasmids using VigoFect (Vigorous) for 72 hours and 6μg HEV replicon RNAs by TransIT-mRNA Transfection Kit (Mirus) for 24 hours. Cells were lysed in ice-cold RIP lysis buffer (50mM Tris pH7.5, 150mM NaCl, 1% tritonX-100, 5% glycerol, supplemented with 1mM DTT, 1mM PMSF, 1/500 P.I. cocktail, 1:100 RNase Inhibitor) for 30min with constant rotation. Add RQ1 DNase into the lysates to digest DNA, and incubate at 37°C for 10min. The lysates were cleared by centrifugation at 13000r/m and 4°C for 15 minutes. 7.5% of the cell lysate was saved as input for RT-qPCR. Cell lysates were aliquoted into two parts. One part was added with 4μg Flag antibody and the other with 4μg IgG, with incubation at 4°C overnight. 25μl equilibrated protein G beads were added for another 4 hours at 4°C. After 4×5 minutes washes by RIP200 buffer (20mM Tris pH7.4, 200mM NaCl, 1mM EDTA, 0.3% TritonX-100, 5% glycerol), RNA was eluted in Proteinase K digestion buffer (50mM Tris 7.4, 150mM NaCl, 0.5% SDS, 5mM EDTA containing 20μg proteinase K) at 55°C for 1 hours. The RNA was extracted by TRIzol Reagent (Invitrogen) according to the manufacturer’s protocol. Reverse transcription was performed using ReverTra Ace qPCR RT Kit (TOYOBO) with random primers. Quantitative real-time PCR was performed using 2×RealStar Green Power Mixture (Genstar) according to the instruction. Primers THU-0158 (5’-TTGCCTCCGAGTTAGTCATC-3’) and THU-0159 (5’-TGCAAAGCATTACCAGACCG-3’) were used for HEV viral genome; Primers THU-0003 (5’-GAAGGTGAAGGTCGGAGTC-3’) and THU-0004 (5’-GAAGATGGTGATGGGATTTC-3’) were used for GAPDH gene amplification for qPCR.

### Negative-strand RNA specific RT-qPCR assay

For HEV negative strand RT-qPCR, we followed the methods published previously [36]. The primer THU-2009 (5’-CGGGAAGGCGACTGGAGTGCCtccgccttggcgaatgctgt-3’) was used to prime reverse transcription and primers THU-0298 (5’-CGGGAAGGCGACTGGAGTGCC-3’) and THU-1240 (5’-cccggcagtactgttctagttc-3’) were used for qPCR assay.

### RNA pull-down assay

RNA pulldown assay was performed as described from previous study with some modifications accordingly [70]. Briefly, biotinylated RNAs were transcribed *in vitro* by HiScribe T7 ARCA mRNA Kit (NEB, E2065S) according to the manufacturer’s protocol. Biotin-16-UTPs (Roche) were added as 10% of UTP in the reactions. About 5 μg of biotinylated RNAs were heated for 5 min at 65°C, then cooled down to room temperature in RNA structure buffer (10 mM Tris–HCl pH 7.0, 100mM KCl, 10 mM MgCl_2_, freshly added 1:100 RNase inhibitor). About 2×10^7^ 293T cells overexpressing ORF1-Flag were used for each RNA pulldown experiment. Cells were trypsin digested and re-suspended in 1 ml lysis buffer (50mM Tris pH7.5, 150mM NaCl, 1% TritonX-100, 5% glycerol, supplemented with 1mM DTT, 1mM PMSF, 1:100 P.I. cocktail, 1:100 RNase Inhibitor) followed by 30 min incubation on rotator. Cell lysates were centrifuged at 13,000 rpm at 4°C for 15 min, and the supernatant was transferred to a new tube. Cell lysates were pre-cleared by 30 μl M280 beads (Life technology) and 20 μg yeast RNA for 1 hour at 4°C, then incubated with 5 μg biotinylated RNA at 4°C overnight, followed by addition of 50 μl equilibrated M280 beads for additional 3 hours at 4°C. After 4×5 min washes by RIP200 buffer (20mM Tris pH7.4, 200mM NaCl, 1mM EDTA, 0.3% TritonX-100, 5% glycerol), proteins bound to RNA were eluted in 2% SDS sample buffer by heating at 95°C for 10 min, and then analyzed by western blot using ORF1 antibody.

### Statistical analysis

Student’s *t* test or one-way analysis of variance (ANOVA) with Tukey’s honestly significant difference (HSD) test was used to test for statistical significance of the differences between the different group parameters. *P* values of less than 0.05 were considered statistically significant.

## Supporting information

S1 Fig Identification of functional *cis*-acting RNA elements by ORF1 transcomplementation system. (A-B) Replication of HEV RNA mutants in ORF1 transcomplemented HepG2C3A cells. A series of 500nt-, 200nt- or 300nt-truncated HEV RNA replicons harboring secretory Gaussia luciferase (Gluc) reporter were transfected into HepG2C3A-ORF1 cells. Cell culture supernatants from each group were collected and Gluc activity measured two days after transfection. The data are presented as the percentage of Gluc activity relative to that of the full-length rHEV-Gluc GAD. Values are means plus standard deviations (SD) (error bars) (n = 3). *, P < 0.05; **, P < 0.01; n.s., not significantly different by one-way ANOVA.
(TIF)

S2 Fig Identification of functional *cis*-acting RNA elements by ORF1 transcomplementation system. (A-B) Replication of series of HEV RNA mutants in ORF1 transcomplemented HepG2C3A cells. Series of 50nt-truncated HEV RNA replicons harboring a secretory Gaussia luciferase (Gluc) reporter were transfected into HepG2C3A-ORF1 cells. Cell culture supernatants from each group were collected and Gluc activity measured two days after transfection. The data are presented as the percentage of Gluc activity relative to that of the full-length rHEV-Gluc GAD. Values are means plus standard deviations (SD) (error bars) (n = 3). *, P < 0.05; **, P < 0.01; ***, P < 0.001; n.s., not significantly different by one-way ANOVA.
(TIF)

S3 Fig Alignment of the *cis*-acting RNA elements across *Orthohepevirus* genus and *Piscihepevirus* genus. (A-B) Sequences of the *cis*-acting RNA elements in ORF1 (A) and ORF2 (B) of *Orthohepevirus A* were not conserved in other *Orthohepevirus* species (*Orthohepevirus B*-*D*) or the *Piscihepevirus* genus. Alignment was performed by MEGA6 software. The conserved sequence is shaded in yellow. G113 and G7335 are highlighted by the arrow.
(TIF)

S4 Fig The function of *cis*-acting RNA elements in multiple HEV genotypes. WT, synonymous mutant (G113C, G113T or G7335A) or GAD mutant replicon RNA of SAR55 (gt1), pSHEV3 (gt3) or TW6196E (gt4) were transfected into HepG2C3A cells. Cell culture medium was collected two days after transfection, and Gaussia luciferase activity was quantified. The numbering denotes the positions of the Kernow C1/p6 viral genome. Values are means plus SD (n = 4). Values are means plus SD (n = 4). ***, P < 0.001 by one-way ANOVA.
(TIF)

S5 Fig ORF1 interaction with the HEV genome. (A) Protein expression of ORF1 synonymous mutants. The WT and synonymous mutants of ORF1 cDNA were cloned into pLVX-IRES-zsGreen vector under the control of a CMV promoter and then transduced into HepG2C3A cells. Immunoblotting assay was performed to determine the expression level of ORF1. (B) A schematic representation of the HEV RNA pull-down procedure to examine the interaction of HEV RNA genome with ORF1 protein. (C) The ORF1 protein associated with HEV WT or mutant genomes was analyzed by immunoblotting assay, and the ORF1 abundance was quantified using ImageJ software. This assay was repeated three times, and the data was pooled as presented in *right* panel. Values are means plus SD (n = 3). ***, P < 0.001, by one-way ANOVA.
(TIF)

S6 Fig The *cis*-acting elements are required for negative-strand HEV RNA synthesis. (A) The 293T cells overexpressing ORF1 (GAD)-Flag were transfected with GAD or synonymous mutants of the negative-strand viral RNA genome (Kernow C1/p6). The cells were lysed and then incubated with Flag antibody to perform the immuno-precipitation assay, with lgG as the negative control. The immune-precipitated complex was subjected to RNA purification, and the ORF1-associated RNA were detected by RT-qPCR analysis. Enrichment of RNA binding to ORF1 is shown as fold change normalized to input. Immunoblotting analysis was performed to confirm the efficacy of ORF1 (GAD)-Flag immunoprecipitation. GAPDH, glyceraldehyde-3-phosphate dehydrogenase. (B) Schematic illustration of the positions of the primers in the negative-strand RNA-specific RT-qPCR assay. For the RT primer (THU-2009), a specific tag sequence was added at the 5′ end for RT to generate cDNA derived from the negative viral RNA genome. The forward (Fwd.) primer (THU-0298) and reverse (Rev.) primer (THU-1240) were used in this study for the qPCR assay. (C) HepG2C3A cells were transfected with the indicated rHEV-Gluc RNA replicon. After 2 days, cells were washed, and intracellular total RNA was extracted and subjected to HEV negative-strand-specific RT-qPCR assay to measure the abundance of antigenome. The data are presented as the percentage of viral negative strand RNA relative to that of the WT. Values are means plus SD (n = 3). *, P < 0.05; **, P < 0.01; n.s., not significantly different by one-way ANOVA.
(TIF)

S7 Fig Prediction of the secondary structure of the *cis*-acting RNA element in the ORF1 coding region. (A) RNA structure prediction of the secondary structures of *cis*-acting RNA elements in ORF1 region 97nt-131nt. RNAalifold [45] (http://rna.tbi.univie.ac.at/cgi-bin/RNAWebSuite/RNAalifold.cgi) was utilized to predict the secondary structures for 97nt-131nt and suggested that G113 resides in a loop region. The *cis*-acting RNA element 102nt-131nt are highlighted in blue. (B) G113C and T116G, G113T and T116G double mutations did not rescue HEV replication. (C) RNA structure prediction of the secondary structures of *cis*-acting RNA elements 6nt-121nt. RNAstructure [47] (http://rna.urmc.rochester.edu) was utilized to predict the secondary structures, indicating that G113 potentially base pairs with U23 in the 5’UTR. The *cis*-acting RNA element is shaded in blue. (D) G113C and T23G, G113T and T23G double mutations did not rescue HEV replication. Values are means plus SD (n = 3). n.s., not significantly different by Student’s t test.
(TIF)
