# Supplementary figures and images for: Identification of functional cis-acting RNA elements in the hepatitis E virus genome required for viral replication
Source: PLoS Pathog. 2020 May 20;16(5):e1008488. doi: 10.1371/journal.ppat.1008488 (PMC7239442; doi:10.1371/journal.ppat.1008488)

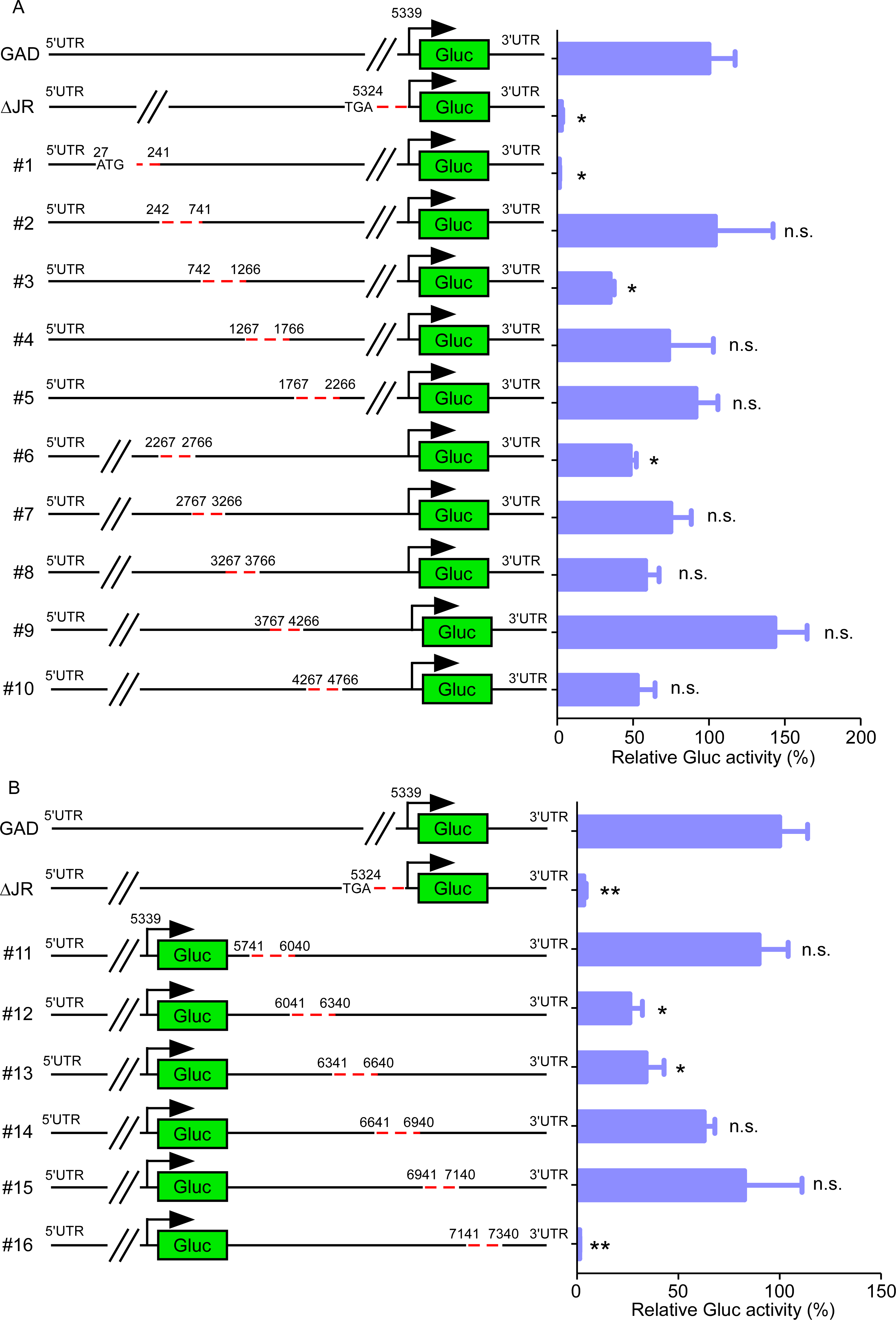

Supplement: S1 Fig — (A-B) Replication of HEV RNA mutants in ORF1 transcomplemented HepG2C3A cells. A series of 500nt-, 200nt- or 300nt-truncated HEV RNA replicons harboring secretory Gaussia luciferase (Gluc) reporter were transfected into HepG2C3A-ORF1 cells. Cell culture supernatants from each group were collected and Gluc activity measured two days after transfection. The data are presented as the percentage of Gluc activity relative to that of the full-length rHEV-Gluc GAD. Values are means plus standard deviations (SD) (error bars) (n = 3). *, P < 0.05; **, P < 0.01; n.s., not significantly different by one-way ANOVA. (TIF) [file ppat.1008488.s001.tif]

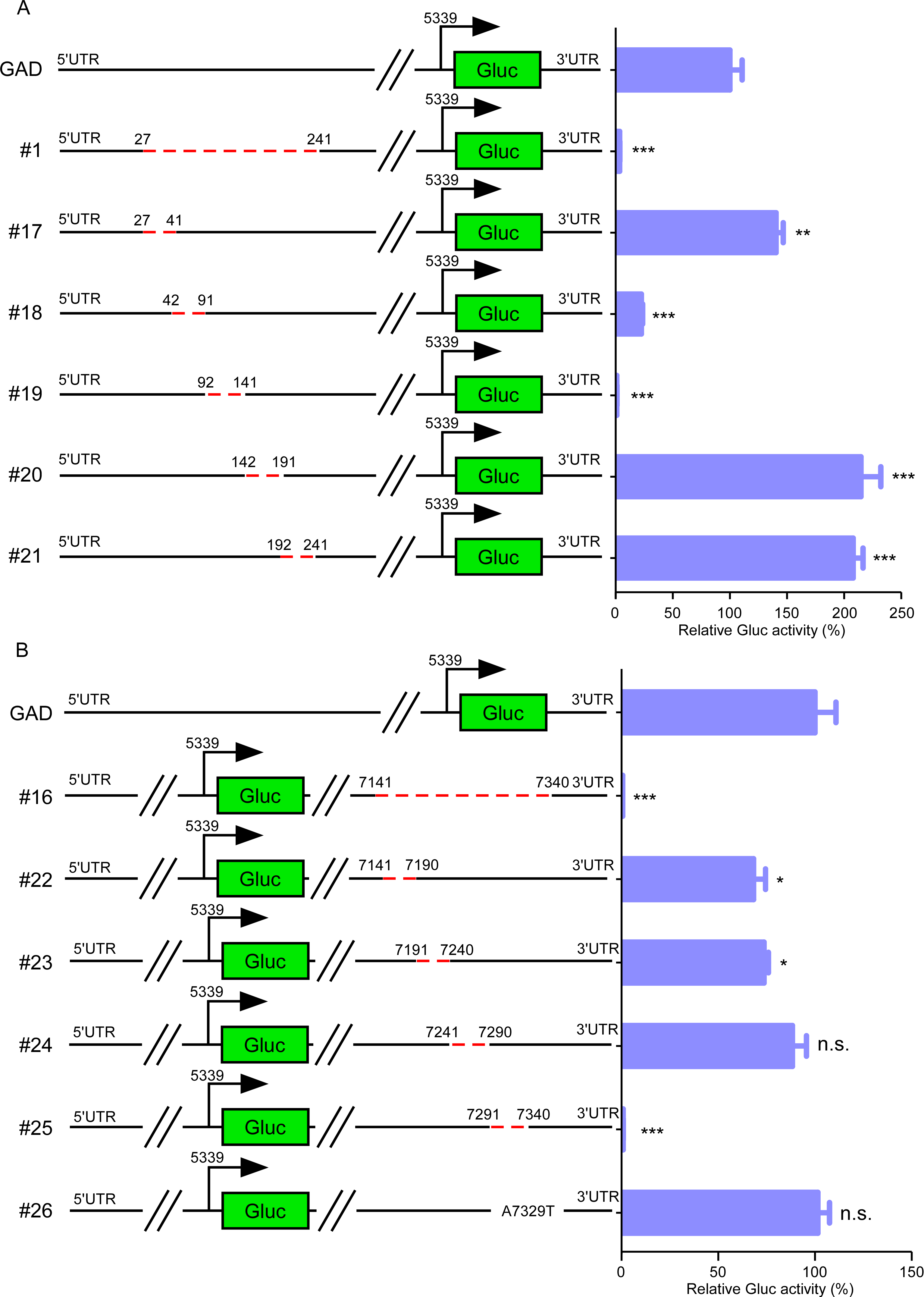

Supplement: S2 Fig — (A-B) Replication of series of HEV RNA mutants in ORF1 transcomplemented HepG2C3A cells. Series of 50nt-truncated HEV RNA replicons harboring a secretory Gaussia luciferase (Gluc) reporter were transfected into HepG2C3A-ORF1 cells. Cell culture supernatants from each group were collected and Gluc activity measured two days after transfection. The data are presented as the percentage of Gluc activity relative to that of the full-length rHEV-Gluc GAD. Values are means plus standard deviations (SD) (error bars) (n = 3). *, P < 0.05; **, P < 0.01; ***, P < 0.001; n.s., not significantly different by one-way ANOVA. (TIF) [file ppat.1008488.s002.tif]

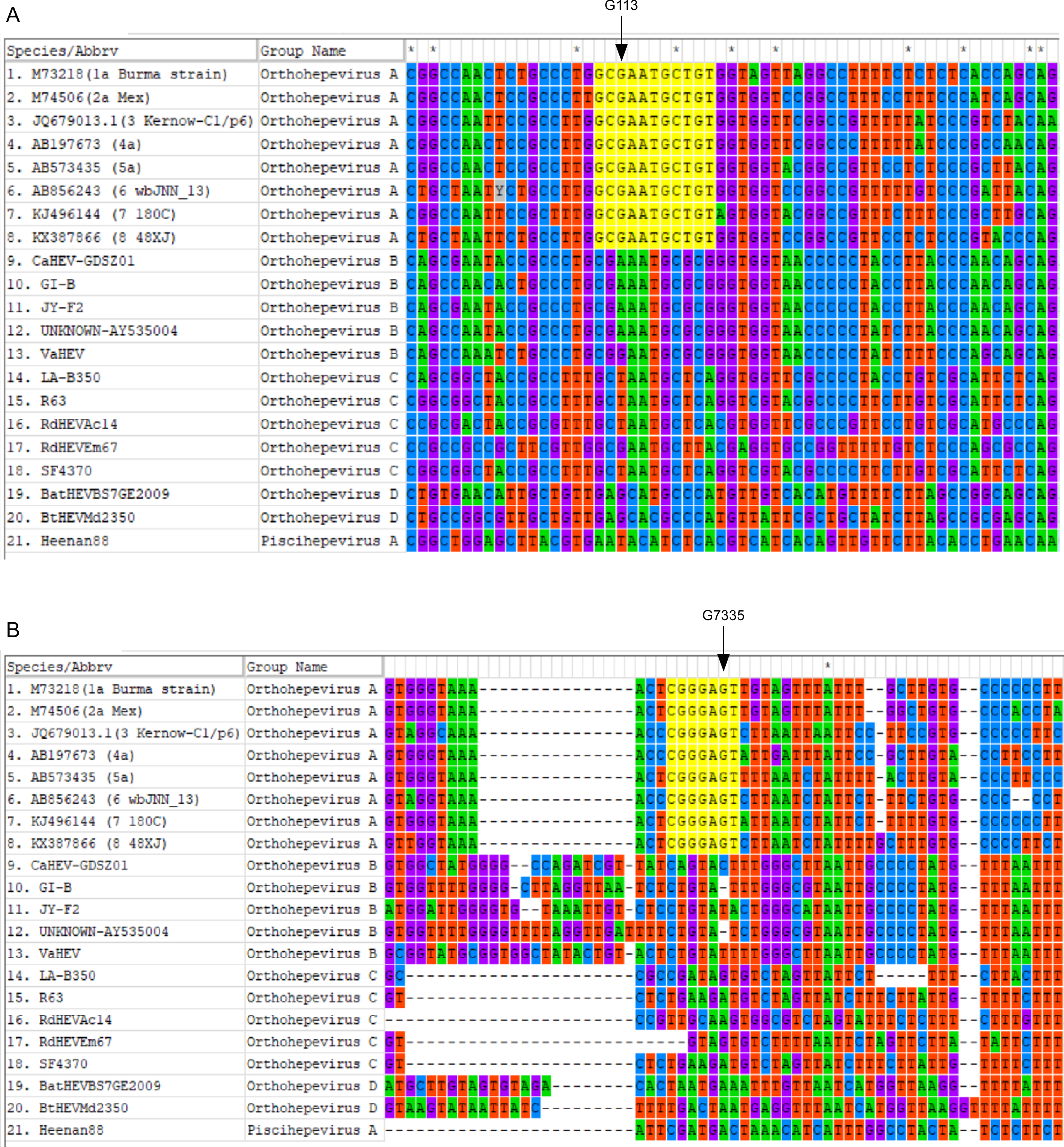

Supplement: S3 Fig — (A-B) Sequences of the cis-acting RNA elements in ORF1 (A) and ORF2 (B) of Orthohepevirus A were not conserved in other Orthohepevirus species (Orthohepevirus B-D) or the Piscihepevirus genus. Alignment was performed by MEGA6 software. The conserved sequence is shaded in yellow. G113 and G7335 are highlighted by the arrow. (TIF) [file ppat.1008488.s003.tif]

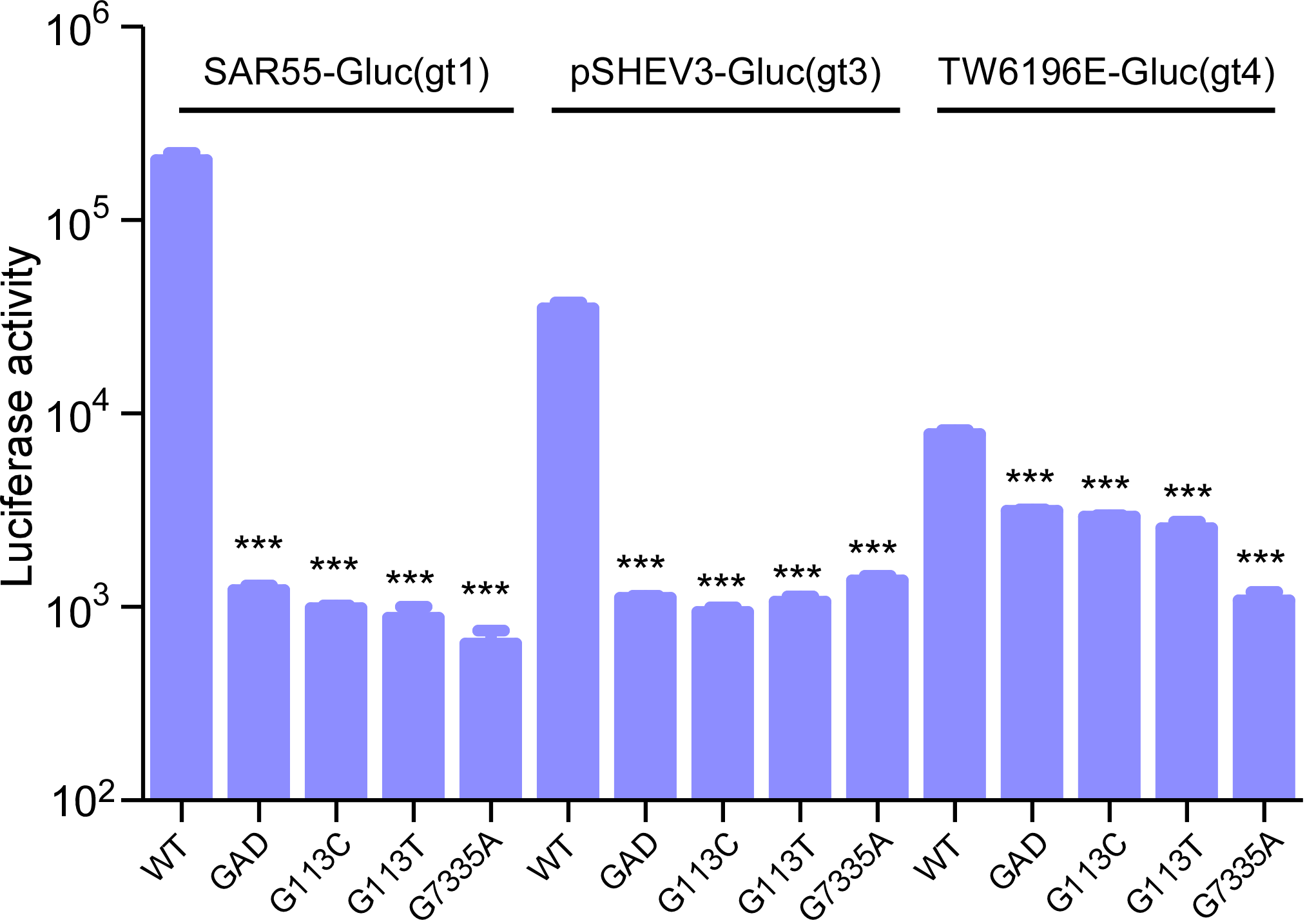

Supplement: S4 Fig — WT, synonymous mutant (G113C, G113T or G7335A) or GAD mutant replicon RNA of SAR55 (gt1), pSHEV3 (gt3) or TW6196E (gt4) were transfected into HepG2C3A cells. Cell culture medium was collected two days after transfection, and Gaussia luciferase activity was quantified. The numbering denotes the positions of the Kernow C1/p6 viral genome. Values are means plus SD (n = 4). Values are means plus SD (n = 4). ***, P < 0.001 by one-way ANOVA. (TIF) [file ppat.1008488.s004.tif]

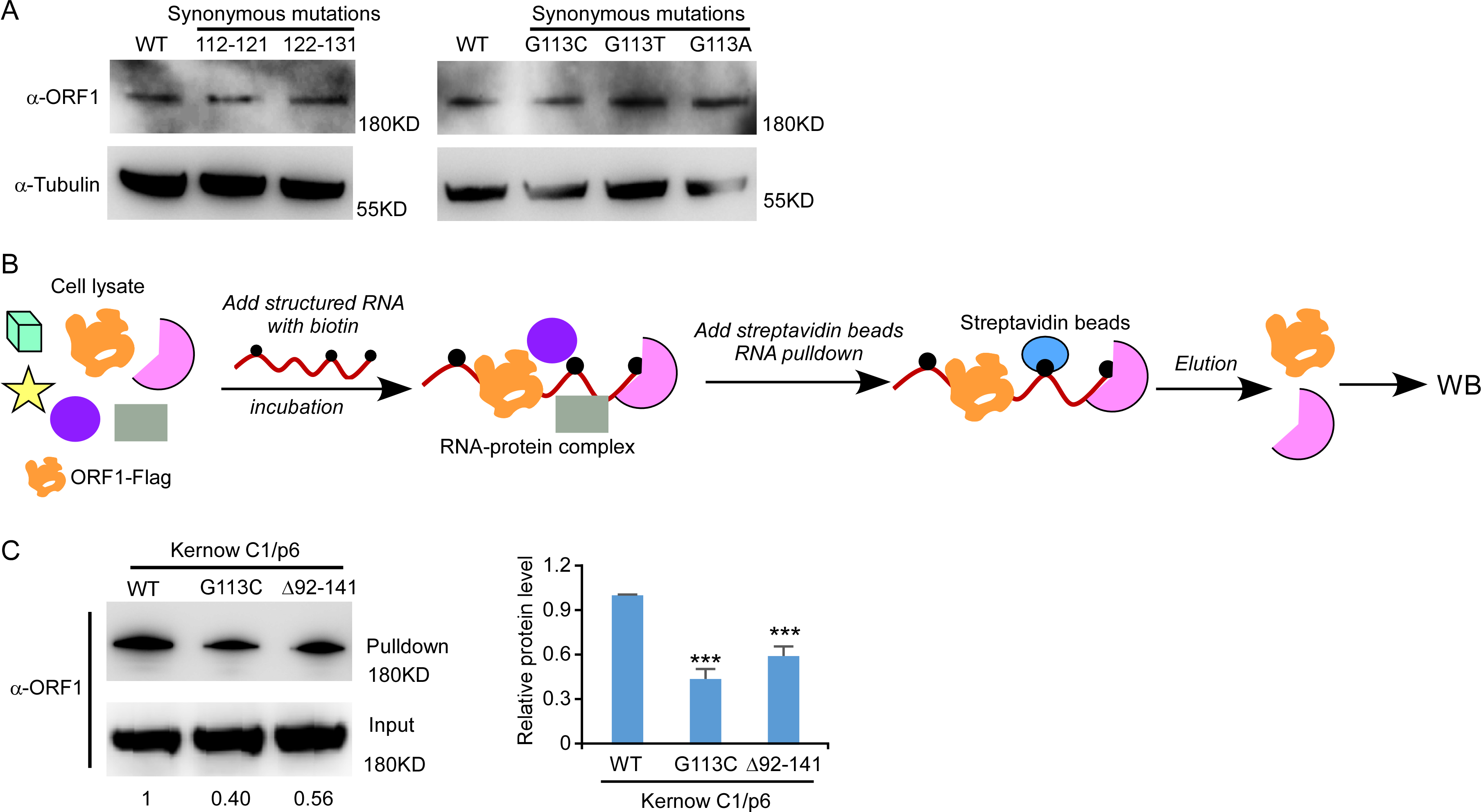

Supplement: S5 Fig — (A) Protein expression of ORF1 synonymous mutants. The WT and synonymous mutants of ORF1 cDNA were cloned into pLVX-IRES-zsGreen vector under the control of a CMV promoter and then transduced into HepG2C3A cells. Immunoblotting assay was performed to determine the expression level of ORF1. (B) A schematic representation of the HEV RNA pull-down procedure to examine the interaction of HEV RNA genome with ORF1 protein. (C) The ORF1 protein associated with HEV WT or mutant genomes was analyzed by immunoblotting assay, and the ORF1 abundance was quantified using ImageJ software. This assay was repeated three times, and the data was pooled as presented in right panel. Values are means plus SD (n = 3). ***, P < 0.001, by one-way ANOVA. (TIF) [file ppat.1008488.s005.tif]

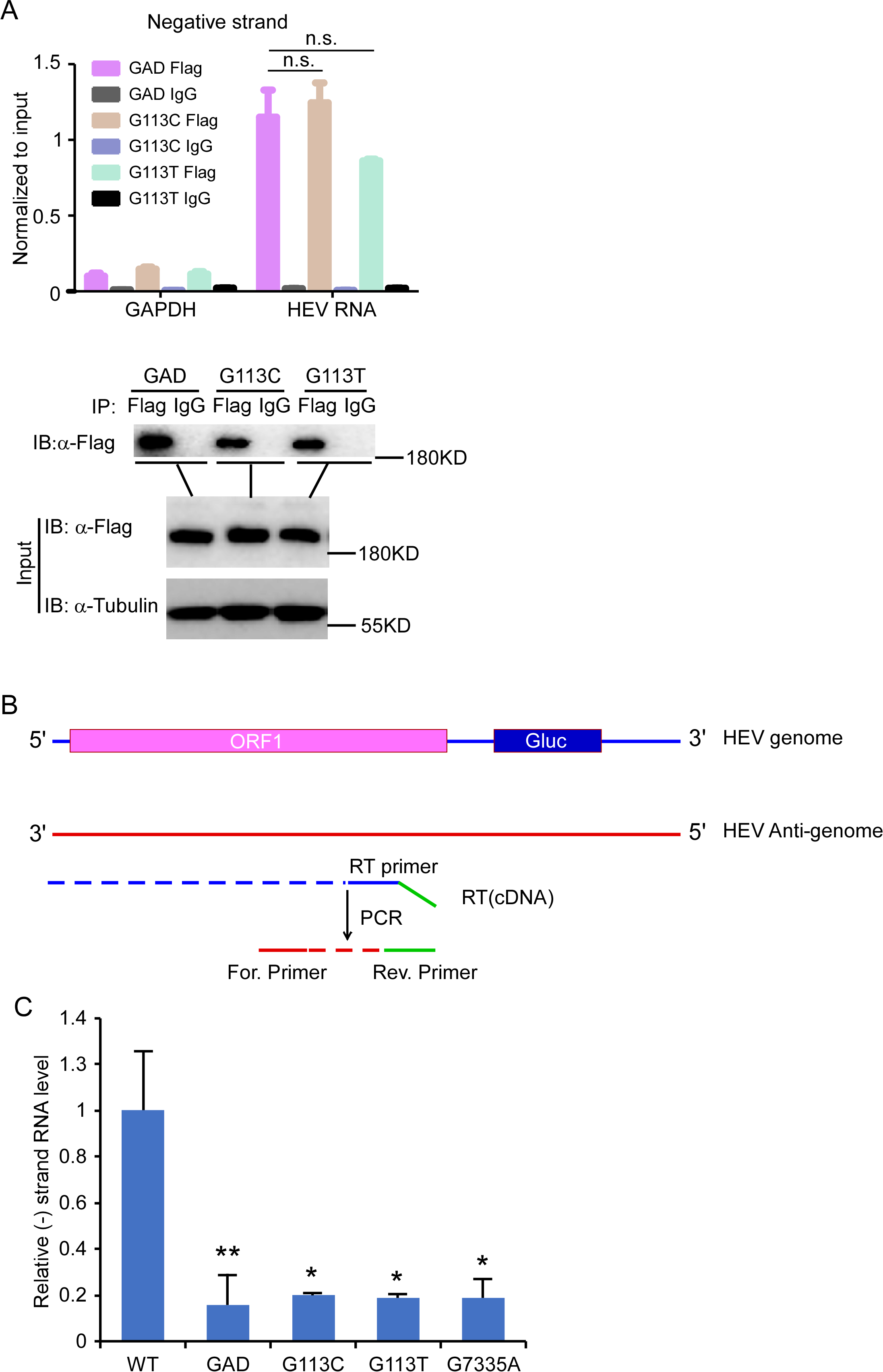

Supplement: S6 Fig — (A) The 293T cells overexpressing ORF1 (GAD)-Flag were transfected with GAD or synonymous mutants of the negative-strand viral RNA genome (Kernow C1/p6). The cells were lysed and then incubated with Flag antibody to perform the immuno-precipitation assay, with lgG as the negative control. The immune-precipitated complex was subjected to RNA purification, and the ORF1-associated RNA were detected by RT-qPCR analysis. Enrichment of RNA binding to ORF1 is shown as fold change normalized to input. Immunoblotting analysis was performed to confirm the efficacy of ORF1 (GAD)-Flag immunoprecipitation. GAPDH, glyceraldehyde-3-phosphate dehydrogenase. (B) Schematic illustration of the positions of the primers in the negative-strand RNA-specific RT-qPCR assay. For the RT primer (THU-2009), a specific tag sequence was added at the 5′ end for RT to generate cDNA derived from the negative viral RNA genome. The forward (Fwd.) primer (THU-0298) and reverse (Rev.) primer (THU-1240) were used in this study for the qPCR assay. (C) HepG2C3A cells were transfected with the indicated rHEV-Gluc RNA replicon. After 2 days, cells were washed, and intracellular total RNA was extracted and subjected to HEV negative-strand-specific RT-qPCR assay to measure the abundance of antigenome. The data are presented as the percentage of viral negative strand RNA relative to that of the WT. Values are means plus SD (n = 3). *, P < 0.05; **, P < 0.01; n.s., not significantly different by one-way ANOVA. (TIF) [file ppat.1008488.s006.tif]

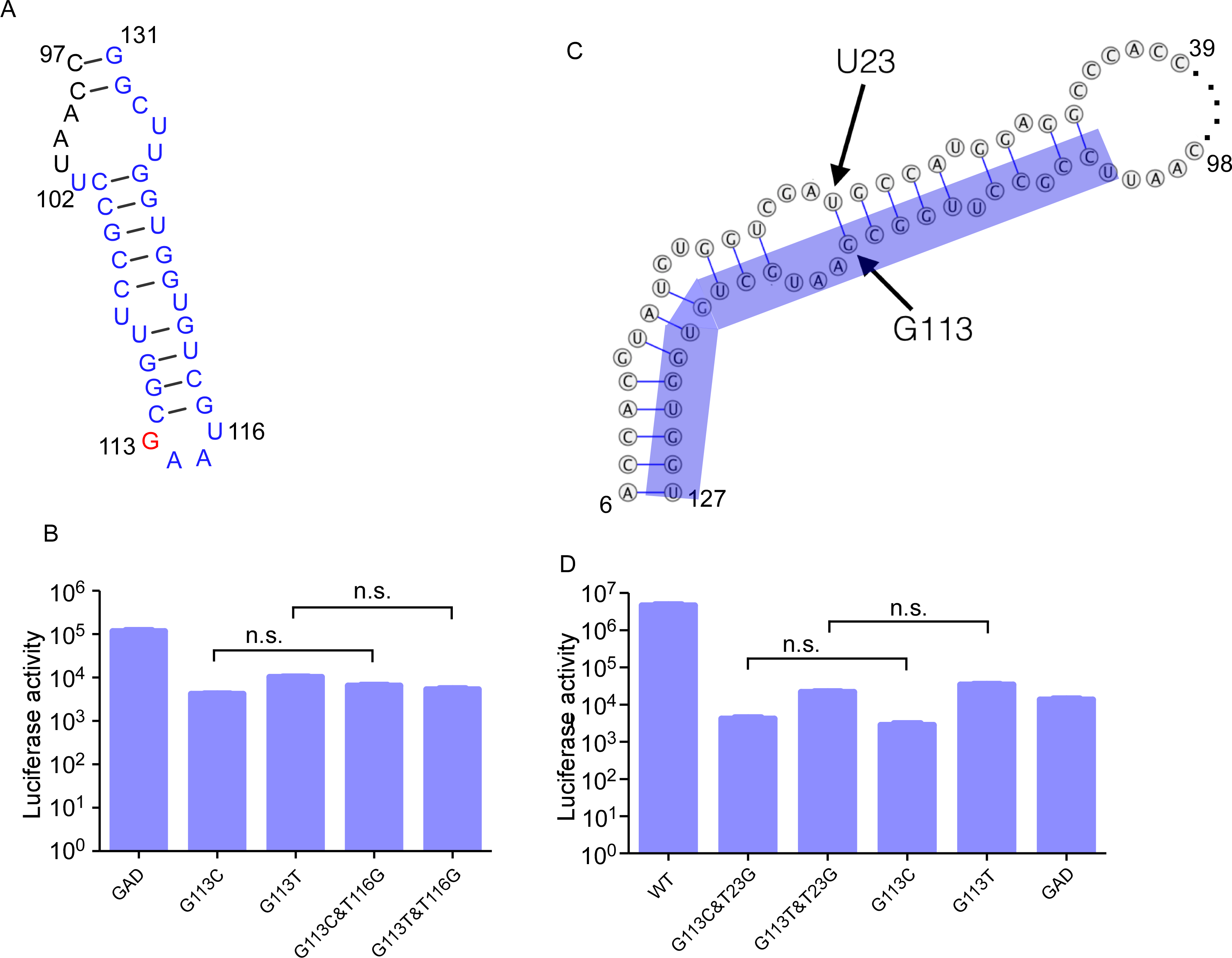

Supplement: S7 Fig — (A) RNA structure prediction of the secondary structures of cis-acting RNA elements in ORF1 region 97nt-131nt. RNAalifold [45] (http://rna.tbi.univie.ac.at/cgi-bin/RNAWebSuite/RNAalifold.cgi) was utilized to predict the secondary structures for 97nt-131nt and suggested that G113 resides in a loop region. The cis-acting RNA element 102nt-131nt are highlighted in blue. (B) G113C and T116G, G113T and T116G double mutations did not rescue HEV replication. (C) RNA structure prediction of the secondary structures of cis-acting RNA elements 6nt-121nt. RNAstructure [47] (http://rna.urmc.rochester.edu) was utilized to predict the secondary structures, indicating that G113 potentially base pairs with U23 in the 5’UTR. The cis-acting RNA element is shaded in blue. (D) G113C and T23G, G113T and T23G double mutations did not rescue HEV replication. Values are means plus SD (n = 3). n.s., not significantly different by Student’s t test. (TIF) [file ppat.1008488.s007.tif]
